# Identification of Unique Gene Expression Profile in Children with Regressive Autism Spectrum Disorder (ASD) and Ileocolitis

**DOI:** 10.1371/journal.pone.0058058

**Published:** 2013-03-08

**Authors:** Stephen J. Walker, John Fortunato, Lenny G. Gonzalez, Arthur Krigsman

**Affiliations:** 1 Wake Forest Institute for Regenerative Medicine, Wake Forest University Health Sciences, Winston Salem, North Carolina, United States of America; 2 Department of Pediatrics Section on Gastroenterology, Wake Forest University Health Sciences, Winston Salem, North Carolina, United States of America; 3 Pediatric Gastroenterology Department, Dr. Miguel Perez Carreño Hospital, Caracas, Venezuela; 4 Pediatric Gastroenterology Resources of New York & Texas, Far Rockaway, New York, United States of America; Temple University School of Medicine, United States of America

## Abstract

Gastrointestinal symptoms are common in children with autism spectrum disorder (ASD) and are often associated with mucosal inflammatory infiltrates of the small and large intestine. Although distinct histologic and immunohistochemical properties of this inflammatory infiltrate have been previously described in this ASD^GI^ group, molecular characterization of these lesions has not been reported. In this study we utilize transcriptome profiling of gastrointestinal mucosal biopsy tissue from ASD^GI^ children and three non-ASD control groups (Crohn's disease, ulcerative colitis, and histologically normal) in an effort to determine if there is a gene expression profile unique to the ASD^GI^ group. Comparison of differentially expressed transcripts between the groups demonstrated that non-pathologic (normal) tissue segregated almost completely from inflamed tissue in all cases. Gene expression profiles in intestinal biopsy tissue from patients with Crohn's disease, ulcerative colitis, and ASD^GI^, while having significant overlap with each other, also showed distinctive features for each group. Taken together, these results demonstrate that ASD^GI^ children have a gastrointestinal mucosal molecular profile that overlaps significantly with known inflammatory bowel disease (IBD), yet has distinctive features that further supports the presence of an ASD-associated IBD variant, or, alternatively, a prodromal phase of typical inflammatory bowel disease. Although we report qPCR confirmation of representative differentially expressed transcripts determined initially by microarray, these findings may be considered preliminary to the extent that they require further confirmation in a validation cohort.

## Introduction

Gastrointestinal (GI) symptoms are common in children with autism spectrum disorders (ASD). Recent studies report an increased frequency of GI symptoms in ASD children compared with typically developing children and those with other developmental delays [1]–[4]. Prospective, controlled studies suggest that as many as 70% of autistic children exhibit chronic GI-related symptoms [1], [5], [6] including diarrhea, laxative-dependent constipation, abdominal distension, failure to thrive, weight loss, feeding problems, and abdominal pain related to extreme irritability, aggression, and self-injury. These symptoms can be minimized or disappear following treatment of the underlying GI disorder [7]. Retrospective chart review studies have shown no increase in GI symptoms in ASD children compared to neurotypical children [8].

In ASD children with GI symptoms who undergo endoscopic and histologic examinations, inflammatory pathology is reported with high frequency [9]–[12]. Features of the GI disease reported originally – ileocolonic lymphoid nodular hyperplasia (LNH) and ileocolitis – have since been expanded to include esophagitis [9], atypical focal gastritis [13], and enteritis [12], [14], [15]. Further analyses of the inflammatory infiltrate in the mucosa and the associated mucosal cytokine profiles have not only confirmed the presence of disease, but suggest characteristic features that distinguish the lesions in ASD children from the more well-described inflammatory bowel diseases (IBDs), i.e. Crohn's disease and ulcerative colitis [10], [13], [14], [16]. In parallel, disturbances in mucosal function [17], [18] and intestinal microflora [19], [20] have been reported and may contribute to the GI pathology in ASD. A recent consensus report regarding GI disorders in individuals with ASDs concluded that ASD children with classic gastrointestinal symptoms often have a chronic inflammatory process “characterized by nodular lymphoid hyperplasia (NLH), enterocolitis, and mucosal infiltration by immune cells along the length of the gastrointestinal tract” [3]. While the clinical significance of these findings is still under investigation, it appears that the immunologic and inflammatory activity in the bowel may be part of a larger, systemic multi-organ immunopathology [21]–[23].

Currently, it is not clear whether the mucosal inflammatory changes seen in ASD^GI^ children represent a milder variant of inflammatory bowel disease or whether a novel pathogenic process is underway. It is possible that a thorough molecular characterization of inflamed gastrointestinal tissue from ASD children and children with established IBD will help to answer this question. Several studies have described the use of gene expression profiling of biopsy-derived gastrointestinal tissue to provide molecular signatures for, and to distinguish between, Crohn's disease and ulcerative colitis [24]–[27]. Using this approach, one group identified a biomarker panel that could be used to distinguish IBD (Crohn's disease (CD) and ulcerative colitis (UC)) from “non-IBD” (in this case irritable bowel syndrome; IBS). The study further identified a subset of transcripts, consisting of seven genes, whose differential expression was useful in distinguishing the IBD subtypes, Crohn's disease and ulcerative colitis, with a high degree of sensitivity and specificity [28]. Gene expression analysis has been recently utilized in the investigation of gastrointestinal dysfunction in ASD children. Building upon prior findings of mucosal brush border enzyme deficiencies in GI symptomatic ASD children, transcript levels of ileal disaccharidases were measured and found to be deficient in those patients [20]. Using pyrosequencing analysis of mucoepithelial bacteria, a significant multi-component dysbiosis in the same ASD cohort was also reported.

Despite the published evidence [9]–[14], [16], the debate still continues [3], [29], [30] as to whether children with ASD and GI symptoms and non-specific mucosal infiltrates have conventionally recognized forms of IBD, a novel IBD phenotype, or no disease at all.

Detailed molecular information, generated from clinical specimens derived from ASD^GI^ children, has the potential to provide valuable clarification of some of these issues. At a minimum, the analysis of differential gene expression in relevant tissue from this group of affected children will lead to a better understanding of the molecular processes involved in their inflammatory disease, including pathways that have been significantly impacted. This in turn may provide a more detailed understanding of the biology that underlies this condition. We report preliminary findings from a large on-going study designed to explore this hypothesis.

The purpose of this study was to compare gene expression profiles (differentially expressed transcripts – DETs) in both ileal and colonic tissues in GI symptomatic ASD children (ASD^GI^) and three non-ASD control groups (Crohn's disease, ulcerative colitis, and normal). The hypotheses being tested were that: (a) DETs in ASD^GI^ distinguish this group from non-inflamed controls, as further evidence of an inflammatory bowel disease (IBD) in the former group; (b) DETs in the Crohn's disease and ulcerative colitis groups also distinguish them from non-inflamed controls; and (c) DETs in ASD^GI^ cases are distinct from those in groups with established IBD.

## Results

### Demographics of Cases and Controls

#### ASD^GI^ samples

A total of twenty five consecutive ASD^GI^ cases (6 *autism*; 19 *autism spectrum disorder*) with histopathologic findings of ileitis, colitis, or both were selected for inclusion in this study (Table 1; Table **S1**). All cases underwent routine diagnostic ileocolonoscopy for chronic gastrointestinal symptoms (Table 2) and demonstrated the histologic presence of ileal infiltrates (ileitis), colonic infiltrates (colitis) or both (ileocolitis). For twenty one of the individuals, both a terminal ileum and a colonic biopsy specimen provided usable RNA. For the remaining four individuals, only a terminal ileum specimen was processed and assayed because the RNA from the corresponding colonic specimens was of insufficient quantity and/or quality.

**Table 1:** Characteristics of Study Population.

Case Status	measure	Age (years)	Gender M(%) F (%)
ASD with GI symptoms (n = 25)	Mean (SD)	5.08 (2.06)	23 (92) 2 (8)
	Range	1.8–10.9	
non-ASD with Crohn's disease (n = 8)	Mean (SD)	12.97 (3.07)	3 (37) 5 (67)
	Range	4–17	
non-ASD with ulcerative colitis (n = 5)	Mean (SD)	12.0 (3.98)	0 (0) 5 (100)
	Range	5–15	
non-ASD – no IBD diagnosis (n = 15)	Mean (SD)	12.2 (3.07)	6 (40) 9 (60)
	Range	6–16	

**Table 2:** Gastrointestinal Symptoms in ASD Study Population.

GI Symptom	ASD^GI^ N = 25 n (%)
Chronic diarrhea	18 (72)
Abdominal pain	14 (56)
Abdominal distention/gas	3 (12)
Constipation	3 (12)
Diarrhea alternating with constipation	2 (8)
Vomiting	2 (8)

### Non-ASD samples

#### Crohn's disease

Eight children with a diagnosis of Crohn's disease were included in this study; seven with active disease (Table 1: Table **S2**). For each individual, a terminal ileum and a colonic biopsy was processed. Pathologic cellular infiltrates were present in either the ileum, colon, or at both locations.

#### Ulcerative colitis

Five children with a diagnosis of ulcerative colitis were included in this study; four with active disease (Table 1; Table **S2**). For each individual, a terminal ileum and a colonic biopsy was processed. Pathologic cellular infiltrates were present in the colon or, in some cases, both ileum and colon.

#### Controls

Fifteen children without identifiable gastrointestinal pathology were included in this study (Table 1; Table **S2**). For each control individual, a terminal ileum and a colonic biopsy specimen was processed. No pathologic infiltrates were seen in either the ileum or colon.

### Principal Component Analysis (PCA)

**Figure 1:**
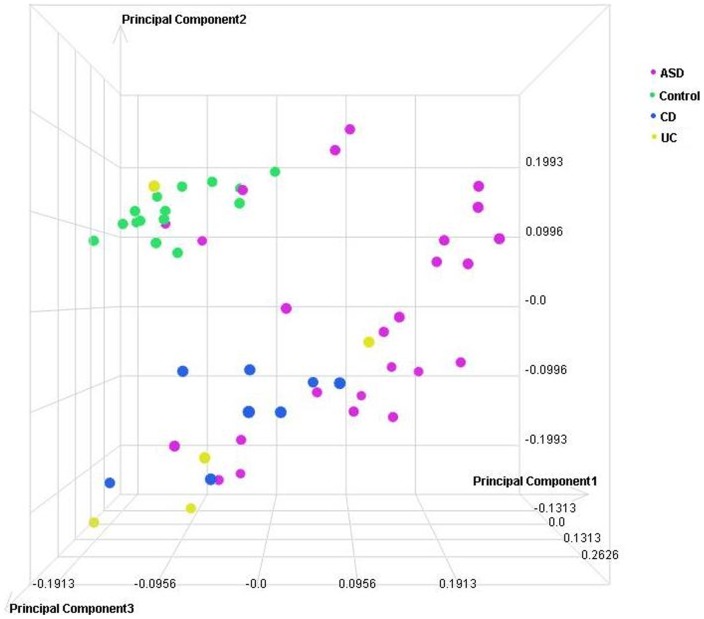
Principal Component Analysis (PCA) scatterplot representing 53 individual microarray datasets from terminal ileum tissues. Each circle represents the cumulative gene expression profile for an individual sample. Samples with similar profiles cluster together in the three-dimensional space.

**Figure 2:**
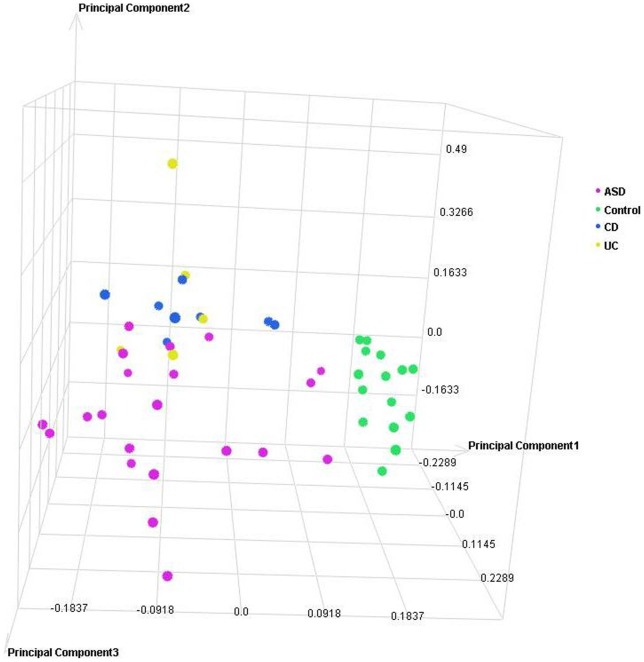
Principal Component Analysis (PCA) scatterplot representing 49 individual microarray datasets from colonic tissues. Each circle represents the cumulative gene expression profile for an individual sample. Samples with similar profiles cluster together in the three-dimensional space.

**Figure 3:**
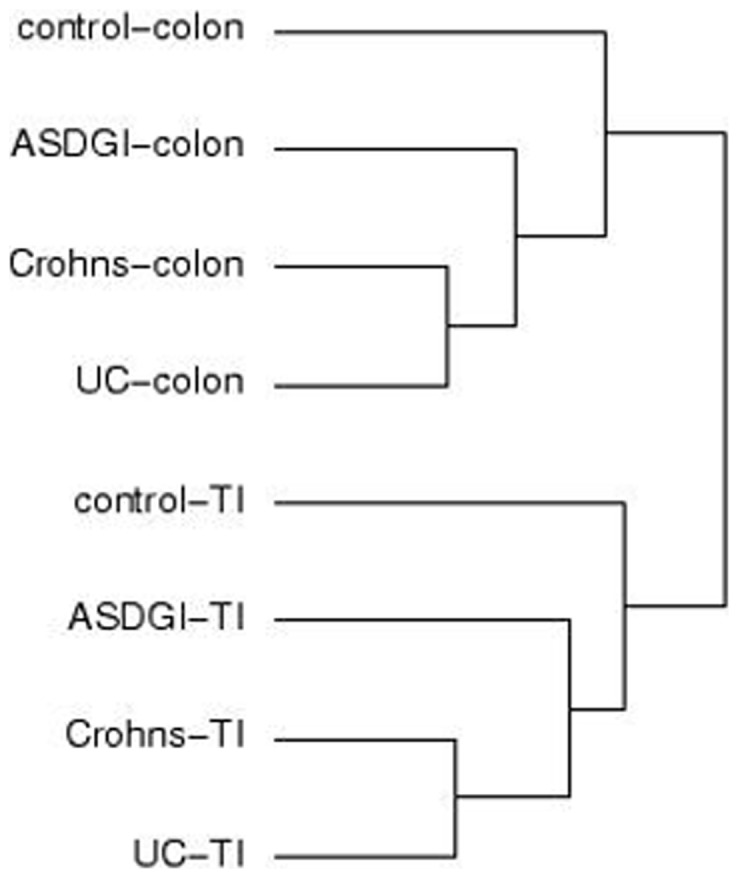
Hierarchial clustering analysis of all samples in all groups. A Kruskal-Wallis test with Benjamini and Hochberg FDR resulted in 5008 DETs (@ fold change ≥2; adjusted p≤0.001) between the 8 groups. In this dendogram, related groups are indicated by the length of the horizontal line (shorter  =  more related), joined by the vertical lines (e.g. in the colon: UC and Crohn's samples are most similar to each other, followed by ASDGI, and then the control group. This pattern is identical in the terminal ileum sample groups).

PCA and unsupervised hierarchical clustering of the sample level data were performed to determine similarity among biological replicates. No filtering was applied to the profile level data prior to PCA. This analysis supports *disease state* as the largest source of variation in these samples (Figures 1 and 2). In an additional analysis of the entire dataset, applying Kruskal-Wallis (@ fold change ≥2; adjusted p≤0.001) with Benjamini and Hochberg FDR, hierarchial clustering demonstrated that: (1) groups of samples cluster by tissue type and, (2) known IBD samples (CD and UC) are more similar to each other than to ASD^GI^ samples (Figure 3).

#### Ileal mucosa gene expression profiles

In the PCA that illustrates findings in ileal mucosa, the 15 control children without identifiable GI disease cluster tightly together while gene expression profiles from inflamed mucosa representing the other groups show broader distribution (variability). Gene expression profiles for the ASD^GI^ samples show the broadest variability in the PCA, suggestive of some potential subgroup(s) (Figure 1). Significant overlap between ASD^GI^ and both Crohn's disease and ulcerative colitis is evident. Gene expression profiles for the Crohn's disease mucosa showed relatively tight clustering that was completely non-overlapping with the histologically normal control group (Figure 1). Interestingly, the majority (80%) of ileal mucosa profiles from children with ulcerative colitis segregated with the profiles for Crohn's disease ileal mucosa.

#### Colonic mucosa gene expression profiles

In the PCA displaying gene expression profiles in colonic mucosa (Figure 2), the 15 control children without identifiable GI disease once again cluster relatively tightly together, and apart from *all* other samples, while inflamed mucosa representing the other groups show broader distribution (variation). Gene expression profiles for the ASD^GI^ samples show the broadest distribution in the PCA (Figure 2). There is some degree of overlap with Crohn's disease and ulcerative colitis but no such overlap with non-inflamed controls. Once again, gene expression profiles for the Crohn's disease mucosa were quite distinct from those in the histologically normal control group. Likewise, for ulcerative colitis mucosa there was considerable overlap with the Crohn's disease profiles but no overlap with histologically normal controls.

### Pairwise Comparisons

Following a determination of the overlap between DETs for three comparisons in each of the two tissues (six comparisons total), each list of DETs unique to a particular comparison (e.g. *ASD^GI^* versus *control* in the terminal ileum) was imported into Ingenuity Pathway Analysis software (IPA: Ingenuity Systems, Inc.; Redwood City, CA) for gene ontology and pathway analysis. The IPA analysis returns significant (p≤0.05) ‘hits’ for each of several categories related to gene networks, biological functions, canonical pathways, and transcription factors. The results that follow focus on findings in the *Diseases and Disorders* (Table 3) and *Physiological System Development and Function* (Table 4) categories, and the *Top Canonical Pathway* (Table 5) involvement category.

**Table 3:** Summary for IPA Diseases and Disorders Category.

Diseases and Disorders	Ileum			colon		
	ASD^GI^	CD	UC	ASD^GI^	CD	UC
						
gastrointestinal disease	*			*	*	
inflammatory bowel disease	*				*	
Colitis	*					
inflammatory response	*	*				
inflammation of organ	*					
antibody response	*					
activation of leukocytes	*					
dermatologic diseases and conditions		*				
Exanthema		*				
Psoriasis		*				
inflammatory disease		*			*	
rheumatic disease		*	*		*	
Dermatitis		*				
cell movement of phagocytes		*				
migration of neutrophils		*				
cardiovascular disease			*			
vascular disease			*			
connective tissue disorder			*			
digestive organ tumor				*		
gastrointestinal tract cancer				*		
colon tumor				*		
neurological disease				*		
Schizophrenia				*		
hyperactive disorder				*		
necrotizing enterocolitis					*	
immunological disease					*	
autoimmune disease					*	
hypersensitive reaction					*	
Sjogren's syndrome					*	
organismal injury and abnormalities						*
Pain						*
Bleeding						*
nutritional disease						*
eating disorder						*
iron deficiency						*
failure to thrive						*

**Table 4:** Summary for IPA Physiologic System Development and Function Category.

Physiologic System Development and Function	ileum			colon		
	ASD^GI^	CD	UC	ASD^GI^	CD	UC
						
humoral immune response	*					
production of antibody	*					
function of B lymphocytes	*					
tissue morphology	*					
quantity of leukocytes	*					
quantity of blood cells	*					
quantity of lymph node cells	*					
morphology of epithelial cells	*					
digestive system development and function	*					
morphology of digestive system	*					
development of gastrointestinal tract	*					
immune cell trafficking		*				
cell movement of myeloid cells		*				
homing of leukocytes		*				
cell-mediated immune response		*			*	
T cell migration		*				
development of Th17 cells		*				
nervous system development and function			*			
long-term potentiation			*			
morphology of nervous tissue			*			
cardiovascular system development and function			*			
migration of endothelial cell line			*			
Angiogenesis			*			
Behavior				*		
social behavior				*		
Learning				*		
Cognition				*		
organ development				*		
growth of intestinal villus				*		
development of brain				*		
lymphoid tissue structure and development					*	
tissue development						*
development of epidermis						*

**Table 5:** Summary for IPA Canonical Pathways Category.

Canonical Pathways	ileum			Colon		
	ASD^GI^	CD	UC	ASD^GI^	CD	UC
						
O-Glycan Biosynthesis	*					
Propanoate Metabolism	*					
Arginine and Proline Metabolism	*					
Alanine and Aspartate Metabolism	*					
Differential Regulation of Cytokine Production in Intestinal Epithelial Cells by IL-17A and IL17F		*				
Differential Regulation of Cytokine Production in Macrophages and T Helper Cells by IL-17A and IL-17F		*				
LXR/RXR Activation		*				
Antigen Presentation Pathway			*		*	
Cysteine Metabolism			*			
B Cell Development			*			
Atherosclerosis Signaling				*	*	*
Factors Promoting Cardiogenesis in Vertebrates				*		
Mitotic Roles of Polo-Like Kinase				*		
T Helper Cell Differentiation					*	
Interferon Signaling					*	
Il-12 Signaling and Production in Macrophages					*	
cAMP-mediated signaling						*
G-Protein Coupled Receptor Signaling						*

### Differentially-Expressed Transcripts in Ileal Mucosa

#### Gene expression profiles in ileal mucosa from ASD^GI^ children compared with histologically normal ileal mucosa from typically developing controls

**Figure 4:**
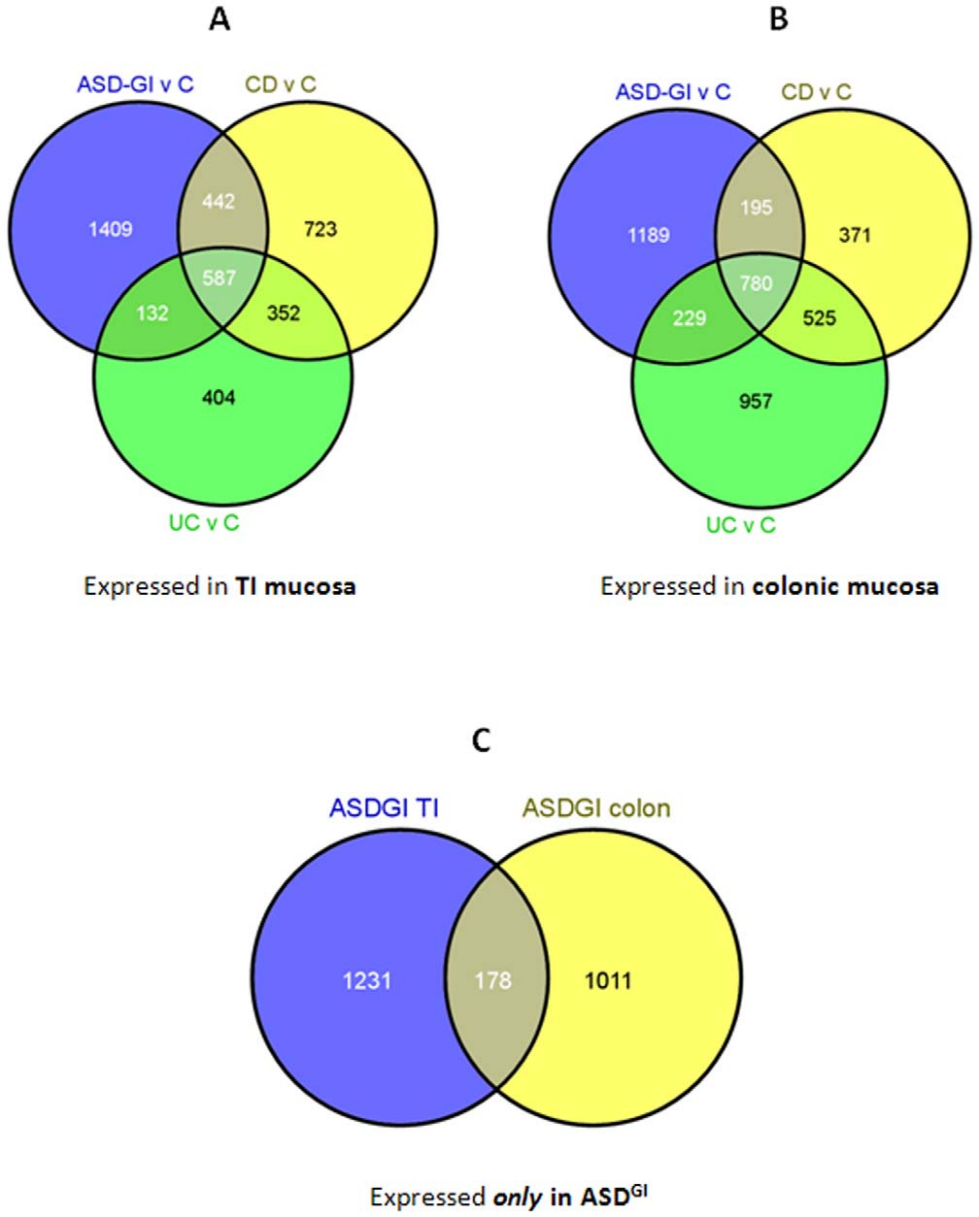
Overlapping unique ASD^GI^ gene expression from TI and colon. Pair-wise comparisons were performed between each of the disease groups (ASD^GI^, CD and UC) and the control (non-histopathologic tissue) samples. **A.** There were 1409 unique DETs (differentially-expressed transcripts) in the ASD^GI^ versus control comparison in TI mucosa. **B**. There were 1189 unique DETs in the ASD^GI^ versus control comparison in colonic mucosa. **C.** The overlap between those two lists is displayed in this Venn diagram. There are a total of 178 DETs shared in ASD^GI^ tissues (Table **S4**). This list of 178 DETs was imported into Ingenuity Pathway Analysis software for further analysis.

Pair-wise analyses between ileal mucosa from ASD^GI^ and non-inflamed control samples resulted in 2570 DETs. Seventy-three percent (1862) of DET's were down-regulated in the ASD group compared with the control group while the remainder were up-regulated (@ fold change ≥2; adjusted p≤0.05). Of these, there were 1409 DETs unique to ASD-GI samples (Figure 4A).

For this list of 1409 DETs unique to the ASD^GI^ ileal biopsies, the *Diseases and Disorders* category returned highly significant associations with: (1) *gastrointestinal disease* [217 genes; p = 1.4×10^−08^] including *inflammatory bowel disease* [42 genes; p = 3.2×10^−05^] and *colitis* [25 genes; p = 8,8×10^−04^] and (2) *inflammatory response* [198 genes; p = 3.8×10^−07^] including *inflammation of organ* [88 genes; p = 3.8×10^−7^], *antibody response* [27 genes; p = 2.1×10^−6^] and *activation of leukocytes* [74 genes; p = 4.9×10^−6^].

The *Physiological System Development and Function* category returned highly significant associations with: (1) *humoral immune response* [91 genes; p = 2.2×10^−10^] including *production of antibody* [52 genes; p = 9.9×10^−10^] and *function of B lymphocytes* [23 genes; p = 4.4×10^−09^]; (2) *tissue morphology* [208 genes; p = 1.8×10^−09^] including *quantity of leukocytes* [109 genes; p = 1.7×10^−8^], *quantity of blood cells* [113 genes; p = 1.4×10^−6^], *quantity of lymph node cells* [8 genes; p = 3.6×10^−4^] and *morphology of epithelial cells* [22 genes; p = 4.5×10^−4^]; and (3) *digestive system development and function* [87 genes; p = 3.1×10^−09^] including *morphology of digestive system* [78 genes; p = 3.1×10^−9^] and *development of gastrointestinal tract* [15 genes; p = 1.8×10^−3^].

Significant numbers of DETs were found in a number of canonical pathways including: *O-Glycan Biosynthesis* [9 genes; p = 1.3×10^−4^], *Propanoate Metabolism* [11 genes; p = 4.6×10^−4^], *Arginine and Proline Metabolism* [13 genes; p = 4.8×10^−4^], and *Alanine and Aspartate Metabolism* [9 genes; p = 5.0×10^−4^].

#### Gene expression profiles in ileal mucosa from typically developing children with Crohn's disease compared with histologically normal ileal mucosa from typically developing controls

Pair-wise analyses between inflamed ileal mucosa from Crohn's Disease samples and non-inflamed control mucosa resulted in 2104 DETs, 71% (1494) which were down-regulated in Crohn's disease mucosa compared to 29% of the DETs that were up-regulated (@ fold change ≥2; adjusted p≤0.05). Of these, there were 723 DETs unique to CD samples (Figure 4A).

The Ingenuity profile for Crohn's disease mucosa versus control mucosa for these unique DETs returned functional category results almost entirely related to inflammation and immune response. The *Diseases and Disorders* category returned a highly a significant association with: (1) *dematologic diseases and conditions* [58 genes; p = 1.8×10^−7^] including *exanthem* [15 genes; p = 1.8×10^−07^] and *psoriasis* [38 genes; p = 3.0×10^−06^]; (2) *inflammatory disease* [82 genes; p = 2.6×10^−7^] including *rheumatic disease* [62 genes; p = 2.6×10^−07^] and *dermatitis* [24 genes; p = 9.7×10^−05^]; and (3) *inflammatory response* [69 genes; p = 4.8×10^−7^] including *cell movement of phagocytes* [34 genes; p = 1.5×10^−06^] and *migration of neutrophils* [12 genes; p = 8.5×10^−06^].

The *Physiological System Development and Function* category returned highly significant associations with: (1) *immune cell trafficking* [57 genes; p = 4.8×10^−7^] including *cell movement of myeloid cells* [33 genes; p = 2.7×10^−06^] and *homing of leukocytes* [24 genes; p = 3.8×10^−5^] and (2) *cell-mediated immune response* [18 genes; p = 7.7×10^−05^] including *T cell migration* [15 genes; p = 4.3×10^−4^] and *development of Th17 cells* [4 genes; p = 6.9×10^−4^].

Significant numbers of DETs were found in several canonical pathways: Differential Regulation of Cytokine Production in Intestinal Epithelial Cells by IL-17A and IL17F [7 genes; p = 9.7×10^−7^], Differential Regulation of Cytokine Production in Macrophages and T Helper Cells by IL-17A and IL-17F [5 genes; p = 6.1×10^−5^], and LXR/RXR Activation [11 genes; p = 2.2×10^−4^].

#### Gene expression profiles in ileal mucosa from typically developing children with ulcerative colitis compared with histologically normal ileal mucosa from typically developing controls

Pair-wise analyses between inflamed ileal mucosa from ulcerative colitis samples and non-inflamed control mucosa resulted in 1475 DETs, 59% (870) which were down-regulated in ulcerative colitis mucosa compared controls while the remainder were up-regulated (@ fold change ≥2; adjusted p≤0.05). Of these, there were 404 DETs unique to UC samples (Figure 4A).

For this analysis, in spite of the comparatively small number of DETs, the *Diseases and Disorders* category returned a highly significant associations with: (1) *cardiovascular disease* [25 genes; p = 7.2×10^−5^] including *vascular disease* [21 genes; p = 5.4×10^−4^] and (2) *connective tissue disorders* [32 genes; p = 6.5×10^−4^] including *rheumatic disease* [29 genes; p = 1.9×10^−03^].

The *Physiological System Development and Function* category returned highly significant associations with: (1) *nervous system development and function* [32 genes; p = 6.4×10^−4^] including *long-term potentiation* [10 genes; p = 6.4×10^−04^] and *morphology of nervous tissue* [16 genes; p = 7.5×10^−3^] and (2) *cardiovascular system development and function* [21 genes; p = 7.4×10^−04^] including *migration of endothelial cell line* [5 genes; p = 7.4×10^−4^] and *angiogenesis* [16 genes; p = 1.8×10^−2^].

Significant numbers of DETs were found in a number of canonical pathways including: *Antigen Presentation Pathway* [5 genes; p = 1×10^−4^], *Cysteine Metabolism* [4 genes; p = 4.7×10^−3^] and *B Cell Development* [3 genes; p = 7.8×10^−3^].

### Differentially-Expressed Transcripts in Colonic Mucosa

#### Gene expression profiles in inflamed colonic mucosa from ASD^GI^ children compared with non-inflamed colonic mucosa from typically developing controls

Pair-wise analyses between inflamed colonic mucosa from ASD^GI^ children and non-inflamed control mucosa resulted in 2393 DETs, 69% (1657) that were down-regulated in ASD^GI^ mucosa compared with those in the control group, while the remainder were up-regulated (@ fold change ≥2; adjusted p≤0.05). Of these, there were 1189 DETs unique to ASD-GI samples (Figure 4B).

For this comparison, the *Diseases and Disorders* category returned highly significant associations with: (1) *gastrointestinal disease* [152 genes; p = 2.4×10^−10^] including *digestive organ tumor* [147 genes; p = 2.4×10^−10^], *gastrointestinal tract cancer* [106 genes; p = 6.5×10^−10^] and *colon tumor* [56 genes; p = 5.6×10^−9^] and (2) *neurological disease* [152 genes; p = 9.3×10^−5^] including *schizophrenia* [50 genes; p = 9.3×10^−5^] and *hyperactive disorder* [16 genes; p = 8.8×10^−4^].

The *Physiological System Development and Function* category returned highly significant associations with: (1) *behavior* [98 genes; p = 4.3×10^−7^] including *social behavior* [8 genes; p = 5.3×10^−3^], *learning* [23 genes; p = 1.4×10^−2^] and *cognition* [25 genes; p = 1.5×10^−02^] and (2) *organ development* [98 genes; p = 6.1×10^−06^] including *growth of intestinal villus* [2 genes; p = 2.5×10^−3^] and *development of brain* [37 genes; p = 1.4×10^−2^].

Significant numbers of DETs were found in a number of canonical pathways including: *Atherosclerosis Signaling* [14 genes; p = 2.4×10^−3^], *Factors Promoting Cardiogenesis in Vertebrates* [11 genes; p = 4.1×10^−3^] and *Mitotic Roles of Polo-Like Kinase* [9 genes; p = 5.5×10^−3^].

#### Gene expression profiles in inflamed colonic mucosa from typically developing children with Crohn's disease compared with non-inflamed colonic mucosa from typically developing controls

Pair-wise analyses between inflamed colonic mucosa from Crohn's disease samples and non-inflamed colonic mucosa from typically developing children resulted in 1871 DETs, 35% (657) which were down-regulated in Crohn's disease mucosa compared with those in the control group while the rest were up-regulated (@ fold change ≥2; adjusted p≤0.05). Of these, there were 371 DETs unique to CD samples (Figure 4B).

The Ingenuity profile for Crohn's disease mucosa versus control colonic mucosa resulted in three highly relevant *Diseases and Disorders* categories that returned several significant associations: (1) *inflammatory disease* [58 genes; p = 1.6×10^−10^] including *rheumatic disease* [47 genes; p = 1.6×10^−10^] and *necrotizing enterocolitis* [2 genes; p = 2.6×10^−3^]; (2) *immunological disease* [53 genes; p = 3.5×10^−9^] including *autoimmune disease* [47 genes; p = 3.5×10^−9^] and *hypersensitive reaction* [15 genes; p = 2.8×10^−3^] and (3) *gastrointestinal disease* [40 genes; p = 1.7×10^−7^] including *Sjogren*'*s syndrome* [11 genes; p = 1.7×10^−7^] and *inflammatory bowel disease* [11 genes; p = 8.4×10^−3^].

The *Physiological System Development and Function* category returned highly significant associations with: (1) *cell-mediated immune response* [21 genes; p = 9.3×10^−6^] and (2) *lymphoid tissue structure and development* [28 genes; p = 9.3×10^−06^].

Significant numbers of DETs are highlighted from five canonical pathways: *Antigen Presentation Pathway* [5 genes; p = 1.3×10^−4^], *T Helper Cell Differentiation* [6 genes; p = 3.3×10^−4^], *Interferon Signaling* [4 genes; p = 1.1×10^−3^], *Atherosclerosis Signaling* [7 genes; p = 1.1×10^−3^] and *Il-12 Signaling and Production in Macrophages* [7 genes; p = 2.2×10^−3^].

#### Gene expression profiles in inflamed colonic mucosa from typically developing children with ulcerative colitis compared with non-inflamed colonic mucosa from typically developing controls

Pair-wise analyses between inflamed colonic mucosa from the ulcerative colitis group and non-inflamed mucosa from the control group resulted in 2491 DETs of which 32% (795) were down-regulated transcripts in the ulcerative colitis group compared with controls (@ fold change ≥2; adjusted p≤0.05) and the majority 68% (1696) were up-regulated. Of these DETs, there were 957 unique to ulcerative colitis samples (Figure 4B).

For this comparison, the *Diseases and Disorders* category returned a highly a significant association with: (1) *organismal injury and abnormalities* [52 genes; p = 5×10^−7^] including *pain* [23 genes; p = 5×10^−7^] and *bleeding* [25 genes; p = 8.8×10^−4^] and (2) *nutritional disease* [53 genes; p = 5×10^−6^] including *eating disorder* [10 genes; p = 3.1×10^−4^], *iron deficiency* [2 genes; p = 4.9×10^−3^] and *failure to thrive* [2 genes; p = 1.2×10^−2^].

The *Physiological System Development and Function* category returned a highly significant association with *tissue development* [94 genes; p = 4.1×10^−7^] including *development of epidermis* [14 genes; p = 6.2×10^−04^].

Significant numbers of DETs were found in a number of canonical pathways including: *cAMP-mediated signaling* [17 genes; p = 1.7×10^−4^], *G-Protein Coupled Receptor Signaling* [27 genes; p = 2.1×10^−3^], and *Atherosclerosis Signaling* [10 genes; p = 2.4×10^−3^].

### Comparison of DETs in ASD^GI^ Sub-groups

#### Differential gene expression unique to tissues from ASD^GI^ children

In order to identify DETs that uniquely occur in the ASD^GI^ tissues, pair-wise comparisons were made between control samples and ASD^GI^ samples in each of the two tissues (terminal ileum and colon; Figure 4A&B). In these two groups of pair-wise comparisons there were 1409 DETs unique to the ASD^GI^ cases in terminal ileum and 1189 DETs unique to the colon in ASD^GI^ cases. The overlap between these two sets of DETs yielded 178 transcripts that are exclusively differentially-expressed in *both* TI and colonic tissues derived from the ASD^GI^ population, but not the others (Figure 4C). When these 178 DETs (Table **S3**) were analyzed using the Ingenuity Pathway Software, three of the top associated biological functions were *inflammatory disease* (7 genes; p = 3.1×10^−3^), *endocrine system development and function* (17 genes; p = 6.6×10^−5^), *and digestive system development and function* (13 genes; p = 2×10^−4^). Significant numbers of DETs were found in a number of metabolic and signaling pathways including: *Granzyme A Signaling* [2 genes; p = 1×10^−2^], *Athersclerosis Signaling* [4 genes; p = 1.6×10^−2^], *Valine, Leucine and Isoleucine Degradation* [3 genes; p = 1.6×10^−2^] and *Clathrin-mediated Endocytosis Signaling* [5 genes; p = 1.7×10^−2^].

A subset of transcripts determined by microarray analysis to be differentially expressed in both TI and colonic tissues in ASG^GI^ cases was verified by quantitative real-time PCR. Twelve transcripts were chosen from the 178 DETs listed in Table **S3** for analysis by PCR. Of these twelve, six transcripts were up-regulated in both the TI and colon, five transcripts were down-regulated in both tissues, and one transcript was up-regulated in colonic tissue but down-regulated in the terminal ileum. Analysis by quantitative PCR confirmed the microarray findings for 11 of 12 transcripts in each of the two tissues (Table 6).

**Table 6:** Comparison of microarray results with qPCR results from 12 representative transcripts differentially-regulated in both terminal ileum and colon, exclusively in ASD^GI^ samples.

TERMINAL ILEUM							
		microarray data			PCR data		
Gene Symbol	Gene Identifier	Ratio	Direction	adj. p-value	Ratio	Direction	p-value
AMPD1	NM_000036	3.7	**↓**	4.47E-07	4.29	**↓**	4.92E-01
IL2RA	NM_000417	2.63	**↑**	2.94E-06	4.93	**↑**	4.10E-05
TXLNG2P	NM_001005852	8.5	**↑**	2.62E-02	97.67	**↑↑**	3.41E-04
RPS4Y1	NM_001008	43.3	**↑↑**	5.89E-03	132.11	**↑↑**	1.46E-03
RPS4Y2	NM_001039567	29.65	**↑↑**	8.41E-03	25.71	**↑↑**	1.02E-03
SCGB2A1	NM_002407	5.25	**↓**	3.64E-07	5.9	**↓**	2.50E-01
ZFY	NM_003411	5.08	**↑**	3.27E-02	1.42	**↑**	1.77E-02
INSL5	NM_005478	2.2	**↓**	2.25E-02	1.27	**↓**	5.27E-01
NTS	NM_006183	5.71	**↓**	7.84E-07	10.33	**↓↓**	3.27E-02
IGF2BP1	NM_006546	4.28	**↑**	1.65E-06	7.13	**↑**	1.22E-01
TNFRSF12A	NM_016639	2.07	**↓**	4.58E-05	1.83	**↓**	9.00E-03
KCTD4	NM_198404	3.58	**↓**	5.23E-06	1.96	**↑**	2.13E-01

COLONIC BIOPSY							
		microarray data			PCR data		
Gene Symbol	Gene Identifier	Ratio	Direction	adj. p-value	Ratio	Direction	p-value
AMPD1	NM_000036	3.85	**↓**	1.62E-04	14.07	**↓↓**	4.37E-04
IL2RA	NM_000417	4.58	**↑**	9.01E-07	3.45	**↑**	1.11E-02
TXLNG2P	NM_001005852	9.71	**↑**	2.60E-02	97.35	**↑↑**	4.18E-02
RPS4Y1	NM_001008	56.76	**↑↑**	1.68E-02	121.79	**↑↑**	1.38E-01
RPS4Y2	NM_001039567	42.65	**↑↑**	1.84E-02	16.05	**↑↑**	3.13E-02
SCGB2A1	NM_002407	4.34	**↓**	6.32E-07	6.4	**↓**	3.18E-03
ZFY	NM_003411	5.45	**↑**	1.84E-02	1.02	**↓**	6.56E-01
INSL5	NM_005478	28.45	**↓↓**	2.15E-04	14.17	**↓↓**	2.20E-01
NTS	NM_006183	2.05	**↓**	2.66E-03	16.92	**↓↓**	5.63E-04
IGF2BP1	NM_006546	14.18	**↑↑**	1.29E-07	7.55	**↑**	1.03E-01
TNFRSF12A	NM_016639	2.94	**↑**	2.11E-03	2.88	**↑**	9.65E-02
KCTD4	NM_198404	4.1	**↓**	2.46E-05	1.4	**↓**	2.20E-01

#### Gene expression profiles in ileal mucosa from ASD children with LNH only (17) compared with ileal mucosa from ASD children with LNH & ileitis (8)

Of the children in the ASD^GI^ group, all 25 had LNH while 8 of the 25 also had histologically confirmed ileitis. A comparison was performed in order to examine the additional effect of ileal inflammation on gene expression within the ASD^GI^ group. Comparison of results from pair-wise analyses between ASD^GI^ cases with LNH only versus ASD^GI^ cases with LNH *and* ileitis resulted in 41 DETs that were unique to the LNH + ileitis group (Table **S4**). When this list of DETs was analyzed in IPA, the *Diseases and Disorders* category returned a highly a significant association with *inflammatory response* [16 genes; p = 5.8×10^−13^], *immunologic disease* [17 genes; p = 1.9×10^−11^] and *dermatological diseases and conditions* [16 genes; p = 2.9×10^−11^]. The *Physiological System Development and Function* category returned a highly significant association with *immune cell trafficking* [13 genes; p = 3.4×10^−14^]. Significant numbers of DETs were highlighted in three key pathways: *IL-17 Signaling* [3 genes; p = 3.1×10^−4^], *Il-17A Signaling in Gastric Cells* [2 genes; p = 9.6×10^−4^] and *Role of IL-17A in Arthritis* [2 genes; p = 4.4×10^−3^].

## Discussion

In these analyses of the gene expression profiles of ASD^GI^ tissue and their comparison to three non-ASD control groups, we have, for the first time, provided molecular characterization of inflamed GI biopsy tissue previously described only in terms of its histologic and immunohistochemical staining properties. Employing differential expression and principle component analysis methodologies we have addressed the three stated hypotheses and found that: (a) DETs in ASD^ GI^ distinguish this group from non-inflamed controls (i.e. non-specific ileocolonic cellular infiltrate in GI symptomatic ASD children is not “normal”), (b) previously published data that demonstrate characteristic DETs in Crohn's disease and ulcerative colitis, as compared to non-inflamed controls, was reaffirmed and, (c) DETs in ASD^ GI^ cases are distinct from both IBD and non-inflamed typically developing controls, though overlap exists between ASD^ GI^ and IBD gene expression profiles.

An analysis of the three groups consisting of inflamed GI tissue demonstrated extensive intergroup DET overlap in both ileum and colon when compared to non-inflamed controls. The finding of overlap between the Crohn's disease and ulcerative colitis samples was expected based on previous comparisons of gene expression in mucosal tissue in IBD patients, however the new findings presented here of DET overlap between ASD^ GI^ and IBD (Crohn's and ulcerative colitis) provides further evidence in support of a novel ASD-associated enterocolitis. Perhaps more importantly, although there were significant numbers of overlapping DETs in each of the two tissues for ASD^ GI^, Crohn's disease, and ulcerative colitis, the pair-wise comparisons between ASD^ GI^ and controls resulted in the largest number of DETs unique to those comparisons (1409 in terminal ileum; 1189 in colonic tissue). This provides important molecular evidence that, while similar to Crohn's disease and ulcerative colitis, gene expression profiles in ASD^ GI^ tissue remain significantly distinct not only from those of known IBD conditions but also from those of non-inflamed tissue as well. It remains to be seen specifically how these molecular (gene expression) differences in Crohn's disease, ulcerative colitis and ASD^GI^ are expressed phenotypically.

In the terminal ileum mucosal tissue, gene expression analysis revealed that for ASD^GI^, Crohn's disease, and ulcerative colitis, a large majority of DETs (59–73%) were down-regulated compared to non-inflamed control tissue. In the pair-wise comparisons for Crohn's disease and for ASD^GI^, the disease categories that were most significantly represented were: (a) gastrointestinal disease and, (b) inflammatory response/disease. In contrast, for ulcerative colitis, disease categories including cardiovascular disease and connective tissue disorders were significantly represented. All of the categories identified within these comparisons were accompanied by highly significant p values, indicating that in all of these tissues there was a strong association with a pathologic gastrointestinal phenotype. The pathway involvement, while demonstrating some common themes across comparisons (e.g. cell signaling and metabolic processes), was somewhat different for each of the three conditions, highlighting variation in the associated underlying biology that distinguishes them.

In the colonic mucosal tissue, gene expression analysis revealed that in CD and UC a majority of the DEGs were up-regulated (65–68%) whereas in ASD^GI^ the majority of transcripts (69%) were down-regulated. The disease categories significantly represented in ASD^GI^ were gastrointestinal disease and neurologic disease. In the CD samples there was a highly significant association with gene ontologies representing inflammatory disease and immunological disease, as well as gastrointestinal disease. The ulcerative colitis profiles were correlated strongly with organismal injury and nutritional disease. The pathways that showed significant involvement in each of the three comparisons again varied somewhat but, as in the terminal ileal mucosa, generally involved cell signaling and metabolic processes.

All of the terminal ileal ASD^GI^ samples displayed lymphoid nodular hyperplasia (LNH) but only a portion of them also had inflammation (ileitis). To determine the additional impact of ileitis on gene expression in ASD^GI^ cases, an analysis was performed between ASD^GI^ TI samples with (8) and without (17) ileitis. Interestingly, the disease categories most significantly represented by these DEGs unique to the LNH + ileitis samples were inflammatory response, immunologic disease and dermatological diseases and conditions. The biological pathways that were found to be significantly regulated in this comparison are largely involved in immune-mediated signaling. This data set adds the additional significant observations that: (1) although the absence of cellular infiltrate does not preclude the presence of a unique molecular signature, the presence of cellular infiltrate (i.e. ileitis) results in further refining the discerning nature of the signature to specific DEGs and, (2) the presence of LNH without ileitis in the ASD^GI^ group is associated with unique DETs, suggesting that LNH in the setting of ASD, chronic gastrointestinal symptoms, and cellular infiltrate (anywhere in the bowel) is part of the disease process.

In summary, the overall gene expression patterns from comparisons of inflamed and non-inflamed tissue in CD, UC and ASD^GI^ exhibited unique DETs as well as some degree of overlap. The dendogram in Figure 3 displays these relationships for each of the two tissues. The Kruskal-Wallis test results lend further support to the findings in the individual pair-wise comparisons; i.e. DETs in IBD conditions (CD and UC) are more similar to each other than to ASD^GI^, and all three groups are more similar to each other than to the non-inflamed controls.

Several aspects of the data are reassuring from a methodological perspective: first, the relatively tight clustering of gene expression profiles from the typically developing non-inflamed group depicted in the PCA for both ileal and colonic mucosa – a clustering that excluded the great majority of those with mucosal inflammation and, second, aberrant gene expression profiles in IBD cases that are in accord with independent reports from other groups [24]–[27]. Abnormal gene expression in ASD^GI^ tissues is consistent with previous reports of cellular and structural changes within the mucosa [11]–[13], [15], [16] accompanied by pro-inflammatory bias in mucosal CD3^+^ lymphocyte cytokine profiles [10], [31], and a more recent report of abnormal mucosal mRNA profiles in other ASD children who, while suffering GI symptoms, did not appear to have associated mucosal inflammation [20]. In the only published study that reports gene expression results in ileal mucosal tissue derived from ASD^GI^ cases compared to controls, Williams et al measured the mRNA levels of three disaccharidases (SI, MGAM and LCT), two glucose transporters (SGLT1 and GLUT2), an enterocyte marker (villin), and a master transcriptional regulator in the intestine (CDX2) [20]. With the exception of villin mRNA (no change), all other transcripts were significantly down-regulated in ASD^GI^ samples compared to controls. Moreover, the ileal expression of the master regulator, CDX2, was a significant predictor of mRNA expression of the three disaccharidases and two transport molecules in ASD^GI^ and Control^GI^ children, based on linear regression models. Data in the present study correlate well with these findings. In the present study, expression of CDX2 was significantly down-regulated in the ASD^GI^ ileum (2.1 fold) as were 2 of the three enzymes (SI and MGAM), both transporters, and villin. These interesting findings may broaden the GI disease repertoire in ASD to include not only mucosal inflammation, as defined by abnormal cellular infiltrate seen during routine light microscopy, but also molecular abnormalities occurring in the absence of obvious light microscopic changes.

One of the study's limitations was the relatively small and unequal sample numbers, especially in the IBD groups, although the pattern of aberrant gene expression was consistent with that described previously for these disease groups. The original goal of this preliminary study was to describe the molecular phenotype(s) for the ASD^GI^ group – the IBD cases were included for comparison. Despite the differences in group sizes, highly significant differences emerged, particularly between ASD^GI^ cases and non-inflamed controls.

Additional factors known to influence human intestinal mucosal gene expression include, but are not limited to, age, gender, ethnicity, prescription medications, diet, and dietary supplements. Insofar as this is a retrospective study designed primarily to explore the relationship between the ASD^GI^ phenotype and inflammatory bowel disease, these potential confounding factors could not be adequately controlled for. The variety of diets, medications, and nutritional supplements in the ASD-GI group is depicted in Table **S1**. For the most part ASD-GI children were on a diet that restricted ingestion of both gluten and casein, and in some instances also soy, whereas individuals in the control groups were not on restrictive diets. In addition, food auto-restriction, a common feature in autism, serves to further limit the variety of foods to which the bowel mucosa is exposed and could potentially impact mucosal gene expression. None of the ASD^GI^ cases in this study were receiving medications known to impact inflammatory processes of the intestinal mucosa for at least four weeks prior to obtaining the biopsies. This includes NSAIDS, H2 blockers, proton pump inhibitors, corticosteroids, antibiotics, probiotics, and immune-suppressants. Well-designed prospective studies that would control for additional mitigating factors such as these are both important and necessary.

Because the cellular composition of biopsy material would be expected to influence gene expression, it is theoretically possible that greater levels of lymphoid tissue present in the younger ASD^GI^ patients in the study group may have contributed to differences between them and the older patients in the other three groups. Although this may have been a factor in the terminal ileal gene expression (all of which demonstrated the histologic presence of LNH), this cannot be the case in the colonic analyses as only two of the colonic specimens contained histologic LNH. It therefore seems likely that LNH, by itself, does not contribute to DET variation in this study.

Lastly, not all study participants were diagnosed with ASD using the same standardized diagnostic tools and by the same clinicians. The variety of diagnostic methods used for participants of this study included the Autism Diagnostic Observation Schedule (ADOS), the Autism Diagnostic Interview-Revised (ADIR), or having met the DSM-IV criteria based on standardized non ASD-specific developmental scales such as the Bayley Scale of Infant and Toddler Development, the Mullen scale of Early Learning, and the Vineland Adaptive Behavior Scales, among others. Some patients were determined to have met DSM-IV criteria without use of a quantitative score on standardized testing.

The relatively broad distribution of gene expression profiles in the ASD^GI^ samples represented in the PCA analysis may be attributable to a number of different factors. First, the larger number of cases in this group, relative to the IBD groups, may account for some of this effect. Heterogeneity in the underlying inflammatory process, its severity and location, as well as the average age of individuals within this group, is among the variables that may influence this distribution. It is also possible that the heterogeneity in gene expression profiles in the ASD^GI^ group is reflective of a disease in various stages of evolution. We may therefore speculate that the broad distribution of DETs in ASD^GI^ is reflective of a dynamic process in which the repertoire of DETs is evolving over time, perhaps towards those of other established IBDs. Longitudinal studies currently underway are designed specifically to address this question.

An important consideration when examining between-group differences in gene expression reported here is the disparity in mean age between the ASD^GI^ group (5 years) and the non-ASD groups (12.5 years). Although this factor represents a limitation to this study, we are not aware of any gene expression studies that have reported a significant age-related pattern of expression in pediatric gastrointestinal tissue. In the absence of these data, we are making the assumption that, while there may be *some* differences in gastrointestinal tissue gene expression in children as they age from 5 to 13, these differences would not significantly skew the overall transcriptome profile. A second important limitation in the design of this study is the lack of gender-matched samples. Given the increased risk of ASD in boys, the ASD cases tend to be mostly male, whereas the non-ASD patients are more evenly distributed between male and female. Once again, although we are not aware of any report suggesting significant gender-related differences in gene expression in pediatric gastrointestinal tissue, we acknowledge that they may exist and if so, could confound the interpretation of these results. Importantly, the largest source of variation found via principal component analysis of gene expression in these 53 biopsy samples was *disease state,* i.e. the presence or absence of light microscopic histopathologic findings.

The IBD patients included in this study represent a spectrum ranging from those presenting at initial diagnosis (not currently on medications) to patients with a flare of symptoms due to previously identified IBD. In many cases, the latter group was maintained on some level of treatment for their IBD. However, in both cases, subjects demonstrated active symptoms justifying the need for endoscopic assessment. Thus, despite the difference regarding immune suppression agents, the vast majority of cases in both patient groups had active disease at the time of endoscopy. The authors acknowledge that the use of medications in IBD patients at the time of procedure represents a limitation of this retrospective study. Future prospective studies will be designed to account for this variability.

Taken as a whole, the picture that emerges is one in which GI symptomatic children with ASD in whom cellular infiltrate is present in the ileum and colon have a distinct molecular signature that is consistent with the larger disease categories of gastrointestinal disease, and more specifically, overlaps with Crohn's disease, ulcerative colitis, and autoimmunity. The shared uniquely expressed DETs seen in both the ileum and colon suggest that intestinal mucosal inflammatory infiltrates in the setting of GI symptomatic patients with ASD reflect a single unifying autoimmune process at play in both the small and large bowel. Future studies are necessary to determine whether these markers can be extended to the larger, non-ileocolitic cohort of ASD children.

## Materials and Methods

This study examined gene expression in histologically inflamed colonic and ileal intestinal mucosal tissue from consecutive GI symptomatic children undergoing diagnostic ileocolonoscopy and biopsy for active GI symptoms. Subjects included children with a diagnosis of ASD (ASD^GI^; n = 25, mean age 5.08±2.06 years; 23 male and 2 female) and three typically-developing groups including: (1) children who underwent diagnostic ileocolonoscopy for chronic GI symptoms in which no histopathology was found (n = 15, mean age 12.2±3.07 years; 6 male and 9 female), (2) children with a diagnosis of Crohn's disease (n = 8, mean age 12.97±3.07 years; 3 male and 5 female), (3) and children with a diagnosis of ulcerative colitis (n = 5, mean age 12.0±4.0 years; all female).

### Case selection and Biopsy Procurement

Most patients of the ASD-GI group (Table 1; Table **S1**) were referred for gastrointestinal evaluation by their primary care provider though some patients were self referred. All were patients of a single pediatric gastroenterologist (AK) and were selected based upon a history of normal development for at least 12 months followed by developmental regression and onset of GI symptoms (Table 2). For all individuals in this group, this was their first diagnostic ileocolonoscopy and no patients were taking medication thought to alter the histologic appearance of the GI mucosa. All cases had ileal lymphoid nodular hyperplasia (LNH) and all had histologically-confirmed colitis and/or ileitis in at least one of 8–10 collected and archived ileocolonic biopsies. 25 consecutive patients meeting these criteria were selected.

All patients were assigned a diagnosis of ASD (Autism, Asperger's or PDD-NOS; 16 had a diagnosis of *autism*; 9 had a diagnosis of *autism spectrum disorder*) by one or more practitioners from the following specialties: pediatric neurology, developmental pediatrics, pediatric psychiatry or psychology. Of the twenty five ASD individuals included in this study, there were 22 Caucasians, one black, one Caucasian/Hispanic, and one whose ethnicity was not recorded. A detailed history of GI symptoms was documented (Table 2). Patients who met clinical criteria for diagnostic ileocolonoscopy and biopsy and whose parents agreed to participate in this IRB-approved study (Copernicus Group Independent Review Board) were provided with a study description and informed written consent from the next of kin, carers or guardians on the behalf of all the minors/children participants involved in all studies was obtained.

Specimens were obtained using a standard disposable forceps biopsy device, in accordance with routine diagnostic biopsy protocol. Immediately upon procurement of biopsy tissue, a specimen from each of seven anatomic locations (from the terminal ileum to rectum) was processed for paraffin embedding and subsequent routine histopathology. Biopsies for microarray analysis were obtained from the divided mucosal specimen at each anatomic location. These tissues were placed directly into RNAlater (Qiagen Inc; Valecia, CA) and stored at −80°C prior to processing.

#### Control biopsy procurement

Prospective controls (Table 1; Table **S2**), recruited through an IRB approved protocol (Wake Forest University Health Sciences Institutional Review Board) from the Pediatric Gastroenterology Clinic at the Wake Forest University Health Sciences, included children who presented with symptoms and laboratory testing suggesting possible intestinal disease (Crohn's disease, ulcerative colitis, celiac disease) or food allergy. Non-IBD Control subjects were further defined as those who, following colonoscopy, were without endoscopic or pathologic findings explaining their symptoms. However, the initial indication for colonoscopy was presence of unexplained GI symptoms ranging from abdominal pain, diarrhea, malnutrition, blood observed in the stools, etc. Failure to diagnose the etiology of observed symptoms by endoscopy was subsequently followed by clinical reassessment or additional diagnostic testing.

No concerns regarding developmental delays for any participant in any of the control groups were reported by parents, relatives, caretakers, or teachers and none were noted by physicians at the Wake Forest Pediatric GI Clinic.

For the twenty eight children in the control groups there were 22 Caucasian, 1 black, and 1 Hispanic. The ethnicities for the other four were not recorded. Tissues for microarray analysis were collected, processed and stored in identical fashion to those from children with ASD. Informed written consent from the next of kin, carers or guardians on the behalf of all the minors/children participants involved in all studies was obtained.

All specimens (cases and controls) were collected and stored in identical fashion (e.g. pinch cold biopsy forceps, immediate placement in RNA later, and freezing at −80 degrees within 24–48 hours), cases were collected at a single location with controls collected at a second location, and both cases and controls were collected using identical specimen collection protocols as outlined in the SOP submissions to the respective IRB's.

### Microarray and Data Analysis

#### Sample preparation

Total RNA was isolated from mucosal biopsies that had been stored in RNAlater by sonicating the tissue in the presence of TriReagent (Molecular Research Center, Inc., Cincinnati, Ohio) according to the method of Chomcynski and Sacchi [32]. Total RNA was purified using RNeasy Minelute Plus columns (includes an on-column DNAse step) and reagents (Qiagen, Valencia, CA) and eluted in nuclease-free water. RNA concentration and quality were determined using a Nanodrop ND-1000 (Nanodrop Technologies, Wilmington, DE) and Agilent Bioanalyzer, respectively. A single biopsy specimen was typically 3–5 mg of tissue and yielded from 3–10 µg of high quality (e.g. RIN ≥ 7) total RNA.

#### Microarray

For microarray hybridizations, 500 ng of total RNA from each biopsy was labeled with fluorescent dye (Cy3; Amersham Biosciences Corp, Piscataway, NJ) using the Low RNA Input Linear Amplification Labeling kit (Agilent Technologies, Palo Alto, CA) following the manufacturer's protocol. The amount and quality of the fluorescently labeled cRNA was assessed using a NanoDrop ND-1000 spectrophotometer and an Agilent Bioanalyzer. According to manufacturer's specifications, 1.6 μg of Cy3-labeled cRNA was hybridized to the Agilent Human Whole Genome Oligo Microarray (Agilent Technologies, Inc., Palo Alto, CA) for 17 hrs, prior to washing and scanning. Data were extracted from scanned images using Agilent's Feature Extraction Software (Agilent Technologies, Inc., Palo Alto, CA).

#### Quantitative Real-time PCR (qPCR)

Quantitative real-time PCR was used to validate representative transcripts that showed differential expression, by microarray, from terminal ileum and colonic tissue in ASD^GI^ tissues compared to control tissues. For these assays, six paired RNAs (one TI and one colonic RNA sample) from ASD^GI^ cases and six paired RNAs from the control individuals were used as representative samples for PCR. Twelve differentially-expressed transcripts were chosen from the 178 DETs listed in Table **S3** for validation. Of these twelve transcripts, six (IL2RA, TXLNG2P, RPS4Y1, RPS4Y2, ZFY, and IGF2BP1) were up-regulated in both the TI and colon, five (AMPD1, SCGB2A1, INSL5, NTS, and KCTD4) were down-regulated in both tissues and one transcript (TNFRSF12A) was up-regulated in colonic tissue and down-regulated in the terminal ileum.

The qPCR analyses were performed using the RT^2^ SYBR Green ROX qPCR Mastermix (Qiagen) with cDNA samples generated from 0.5 ug total RNA using RT^2^ First Strand Kit (Qiagen) following the manufacturer's instructions. Custom-made 96 well plates (SABiosciences) contained primers for each of the 12 transcripts of interest, two reference genes (ACTB and GAPDH) and two positive controls, aligned in two adjacent columns on the plate. Each 96 well plate was used to assay six individual cDNAs. Following the qPCR run on a StepOnePlus Real-time PCR system (Applied Biosystems), using the custom plate manufacuturer's recommendations, the raw data from each 96 well plate (4 total) were uploaded to the SABiosciences web-based analysis software to determine differential expression parameters (fold change and p value). The software automatically performs all ΔΔC_t_ based fold-change calculations from the uploaded raw quantification cycle data. The results from these assays, compared to the corresponding microarray results, can be found in Table 6.

### Data Analysis

#### Pair-wise analysis to determine differentially-expressed transcripts

Gene expression data were uploaded into the GeneSifter® Analysis Edition (Geospiza, Inc, Seattle, WA) software suite. For all pair-wise comparisons the data were first normalized by global normalization using the median intensity and transformed (log base 2) prior to running the non-parametric Wilcoxon Rank-Sum Test, with Benjamini-Hochberg FDR correction, to generate lists of differentially-expressed transcripts (DETs). The fold-change threshold was set at 2.0 and the data were considered significant if the comparison had an associated log ratio adjusted p-value less than 0.05. The list of differentially-expressed transcripts from a given pair-wise comparison was then imported into Ingenuity Pathway Analysis software for determination of biologically relevant functional categories and canonical pathway involvement. This analysis was performed for each of the eight individual pair-wise comparisons.

#### Principal component analysis

In order to determine the overall similarity between samples, ratio data were subjected to Principal Components Analysis (PCA) and two-way agglomerative cluster analysis using Ward's minimum variance as heuristic criteria and Pearson correlation as the distance metric for *experiments*, and average linkage as heuristic and Pearson correlation distance as the distance metric for *genes* to determine the overall similarity between samples and within groups (Rosetta Resolver version 7.2.2.0). No filtering was applied to the profile level data prior to PCA.

#### Hierarchial clustering analysis

The non-parametric Kruskal-Wallis test, with Benjamini and Hochberg FDR correction, was performed on all 102 microarray datasets (@ fold change ≥2; adjusted p≤0.001) representing the eight experimental groups (four conditions; two tissues).

## Supporting Information

Table S1 **Demographic and medical history data for ASD^GI^ cases.**
(XLSX)

Table S2 **Demographic and medical history data for non-ASD controls.**
(XLSX)

Table S3 **Gene list for 178 transcripts that were differentially-regulated in both terminal ileum and colon, exclusively in ASD^GI^ samples.**
(XLSX)

Table S4 **Gene list for 41 transcripts that were differentially-regulated in a comparison of ASD^GI^ samples with LNH + ileitis.**
(XLSX)
